# The Relationship between Simple Snoring and Metabolic Syndrome: A Cross-Sectional Study

**DOI:** 10.1155/2019/9578391

**Published:** 2019-04-10

**Authors:** Juanjuan Zou, Fan Song, Huajun Xu, Yiqun Fu, Yunyan Xia, Yingjun Qian, Jianyin Zou, Suru Liu, Fang Fang, Lili Meng, Hongliang Yi, Jian Guan, Huaming Zhu, Bin Chen, Shankai Yin

**Affiliations:** ^1^Department of Otolaryngology-Head and Neck Surgery, Shanghai Jiao Tong University Affiliated Sixth People's Hospital, Shanghai 200233, China; ^2^Shanghai Key Laboratory of Sleep Disordered Breathing, Shanghai 200233, China

## Abstract

**Purpose:**

This cross-sectional study was performed to assess the relationship between simple snoring and metabolic syndrome (MetS).

**Methods:**

A total of 5635 participants including 300 healthy volunteers without snoring allegedly were initially included from 2007 to 2016. Polysomnographic variables, anthropometric measurements, and biochemical indicators were collected. The polynomial linear trend test was used to assess the linear trend across snoring intensity for metabolic score, and logistic regression was used to evaluate the odds ratios (ORs) for MetS after controlling for age, sex, obesity, smoking status, and alcohol consumption.

**Results:**

The final study population consisted of 866 participants. Simple snorers showed more severe metabolic disorders and higher prevalence of MetS than nonsnorers. A significant linear trend was observed between snoring intensity and metabolic score. Simple snoring was significantly associated with increased odds for MetS among all participants (OR = 2.328, 95% CI: 1.340–4.045) and female participants (OR = 2.382, 95% CI: 1.136–4.994) after multivariable adjustment. With regard to MetS components, simple snoring was significantly associated with increased odds for hypertension (OR = 1.730, 95% CI: 1.130–2.650), abdominal obesity (OR = 1.810, 95% CI: 1.063–3.083), and hyper-triglycerides (TG) (OR = 1.814, 95% CI: 1.097–2.998) among all participants, with hypertension (OR = 3.493, 95% CI: 1.748–6.979) among males and with abdominal obesity (OR = 2.306, 95% CI: 1.245–4.270) and hyper-TG (OR = 2.803, 95% CI: 1.146–6.856) among females after multivariable adjustment.

**Conclusions:**

After excluding the influence of repeated apnea and hypoxia, simple snoring was still significantly associated with MetS, especially in women. Furthermore, the associations were more obvious for hypertension among males and for abdominal obesity and hyper-TG among females. In addition to OSA, simple snoring also should be valued.

## 1. Introduction

Snoring is commonly described as a coarse and vibratory sound during sleep resulting from partial obstruction of inspiration in the oropharynx [1]. The prevalence of snoring varies from 2% to 85% [1–3]. Simple snoring may represent the beginning of a sleep-disordered breathing (SDB) continuum, which ranges from partial airway collapse and mildly increased upper airway resistance to complete airway collapse and severe obstructive sleep apnea (OSA) lasting for 60 s or more [2, 4]. There is accumulating evidence that snoring is associated with several health problems, including sleepiness, cardiovascular diseases, metabolic syndrome (MetS), and all-cause mortality [5–7].

MetS, a combination of excess abdominal obesity, dyslipidemia, hypertension, hyperglycemia, and insulin resistance (IR) [8], is related to increased risk of cardiovascular events and mortality [9]. Previous studies have demonstrated a relationship between snoring and MetS, but patients with OSA were not excluded from the study populations [10–12]. Such association between snoring and MetS may be mediated by OSA, as heavy snoring is always accompanied with sleep apnea [3], and both snoring and sleep apnea are related to mechanical obstruction of the upper airway [13]. However, most common snorers do not have OSA [4]. Therefore, further research is required to determine whether simple snoring itself, as a more common disease, is independently related to increased odds for MetS and its components.

To exclude the effects of more severe sleep apnea, we conducted a cross-sectional study among non-OSA participants to examine whether simple snoring itself is associated with increased odds for MetS and metabolic disorders, such as obesity, hypertension, dyslipidemia, and IR.

## 2. Methods

**Figure 1:**
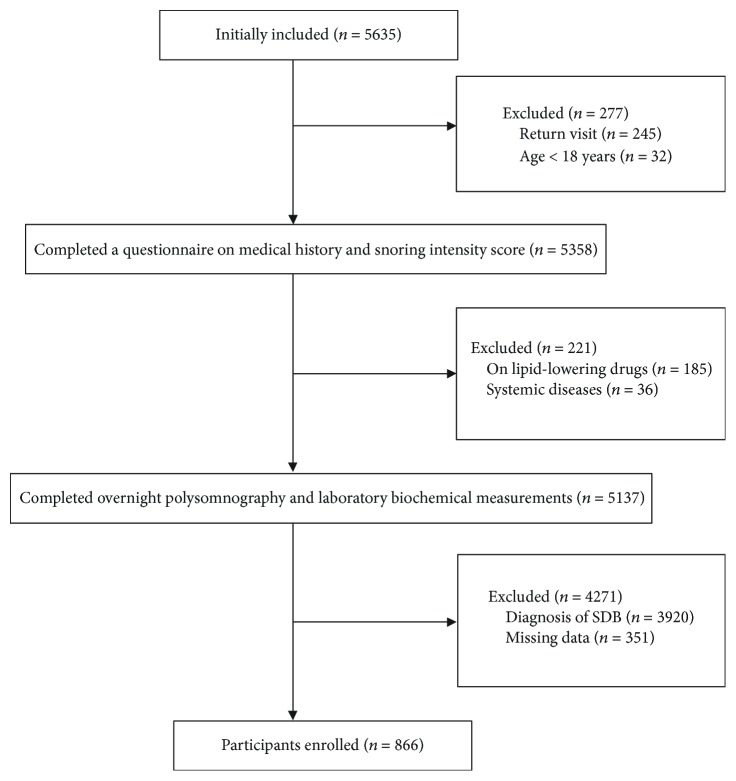
Flow chart of study population enrollment.

A total of 5635 participants were initially included in the study. Among them, 300 were healthy volunteers specially recruited without snoring allegedly; the other participants were patients referring to the sleep center of Shanghai Jiao Tong University Affiliated Sixth People's Hospital for suspected SDB from 2007 to 2016. They were mainly from cities in southeastern China and all completed surveys regarding smoking habits, alcohol consumption, and medical history. 4769 participants were excluded for the following reasons: return visit; age < 18 years; taking lipid-reducing medications prior to the study, which could affect the serum lipid profiles' levels; various systemic diseases (i.e., malignancy, chronic kidney disease, and unstable cardiopulmonary diseases, such as congestive heart failure or intrinsic pulmonary disease); diagnosis of SDB; and missing data (lacking information of smoking, drinking, lipid-lowering medication taking, etc.). Finally, a total of 866 participants including 187 nonsnorers and 679 simple snorers were enrolled in the analysis (Figure 1). Written informed consent was obtained from each participant according to the guidelines outlined by the National Ethics Regulation Committee. This study was approved by the Internal Review Board of the Institutional Ethics Committee of Shanghai Jiao Tong University Affiliated Sixth People's Hospital and was conducted in accordance with the tenets of the Declaration of Helsinki.

### 2.1. Anthropometric and Metabolic Measurements

All measurements were performed using the standard methods mentioned in our previous research [14], with the participants dressed in lightweight clothing and with bare feet. Body mass index (BMI) was calculated as body mass in kilograms divided by the square of the patient's height in meters. Neck circumference (NC) was measured at the level of laryngeal prominence, waist circumference (WC) in the middle between the 12th rib and the iliac crest, and hip circumference (HC) at the level of the anterior superior iliac spine at the broadest circumference below the waist using a measuring tape. The waist-to-hip ratio (WHR) was determined as WC (cm)/HC (cm). In accordance with the guidelines of the American Society of Hypertension [15], blood pressure was measured at approximately 08:00 with patients in a seated position using a mercury sphygmomanometer after a 5 min rest. It was recorded as the mean of three measurements taken at 1 min intervals.

A fasting blood sample was taken from the antecubital vein of each patient in the morning after polysomnographic monitoring. Fasting serum glucose and lipid profiles were measured in the hospital laboratory using routine procedures. Serum lipid profiles included total cholesterol (TC), triglycerides (TG), high-density lipoprotein (HDL), low-density lipoprotein (LDL), apolipoprotein A-I (apoA-I), apolipoprotein B (apoB), apolipoprotein E (apoE), and lipoprotein(a) (Lpa) (Hitachi, Tokyo, Japan). An immunoradiological method was used to measure the fasting serum insulin level. Insulin sensitivity was evaluated using the homeostasis model assessment of insulin resistance (HOMA-IR): HOMA-IR = fasting glucose (mmol/L) × fasting insulin(*μ*U/mL)/22.5 [16].

### 2.2. Polysomnography

Overnight polysomnography (PSG) (Alice 4 or 5, Philips Respironics, Pittsburgh, PA) was performed from 22:00 to 06:00 according to the criteria of the American Academy of Sleep Medicine [17], including electroencephalogram (EEG), left and right electrooculogram (EOG), genioglossus electromyogram, electrocardiogram (ECG), pulse oxygen saturation, nose and mouth airflow, thoracic-abdominal movement, and body position. Apnea was defined as the complete cessation of airflow lasting for at least 10 s. Hypopnea was defined as a ≥50% reduction in airflow for at least 10 s with a decrease in oxyhemoglobin saturation of ≥3% or a ≥30% reduction in airflow for at least 10 s with a decrease in oxyhemoglobin saturation of ≥4%. The apnea hypopnea index (AHI) was defined as the number of apnea and hypopnea events per hour during sleep. The diagnosis of OSA was determined by AHI, and an AHI ≥5 events/h was defined as OSA [17].

### 2.3. Snoring Assessment

As snoring sounds are commonly described as a nuisance by the bed partner of the affected individual, information on snoring was collected from a bed partner or family member. Snoring intensity was evaluated using a 10 cm visual analogue scale (VAS) from 0 to 10: 0 represents no snoring, 1–3 represents minimally annoying, 4–6 represents moderately annoying, 7–9 represents annoying, and 10 represents extremely annoying [18]. We defined 0 as no snoring, 1–3 as mild snoring, 4–6 as moderate snoring, and 7–9 as severe snoring. Besides, in order to ensure a more even distribution for analysis, we incorporated 10 into the severe snoring group.

### 2.4. Metabolic Score

MetS was defined according to the NCEP ATP III criteria with the modified WC criteria for Asians [19] as the presence of at least three of the following five clinical features: (1) elevated WC: ≥90 cm in men and ≥80 cm in women; (2) elevated TG: ≥1.70 mmol/L; (3) reduced HDL <1.03 mmol/L in men and <1.30 mmol/L in women; (4) elevated blood pressure: SBP ≥130 mmHg or DBP ≥85 mmHg or on antihypertensive drug treatment in a patient with a history of hypertension; and (5) elevated fasting glucose ≥5.6 mmol/L or on drug treatment for elevated glucose. A metabolic score was established as the total number of positive diagnostic criteria of metabolic syndrome in each participant [14].

### 2.5. Statistical Analysis

All statistical analyses were performed using SPSS (version 23.0; SPSS Inc., Chicago, IL). All values were examined for normal distribution prior to statistical analysis. Data are presented as the median (interquartile range [IQR]), mean ± standard deviation (SD), or *n* (%) if they are skewed, normally distributed, or categorical. Normally distributed or skewed variables were analyzed using the independent samples *t*-test or Mann–Whitney *U* test, respectively. Categorical variables were analyzed using the chi-square test or Fisher's exact test. The polynomial linear trend test was used to assess the linear trends across snoring intensity for metabolic score. Independent associations between snoring and MetS and its components were analyzed, using multivariable logistic regression after adjusting for relevant covariates. Odds ratios (ORs) are presented with 95% confidence intervals (CIs). In all analyses, *P* < 0.05 was taken to indicate statistical significance.

## 3. Results

### 3.1. Basic Characteristics

The 866 participants were divided according to snoring intensity into the simple snoring group (*n* = 679) and nonsnoring group (*n* = 187). The proportion of males was greater in the simple snoring group than in the nonsnoring group (63.0% vs. 41.7%, respectively, *P* < 0.001). Compared to the nonsnoring group, simple snorers were more obese (evidenced by higher BMI, NC, WC, HC, and WHR, *P* < 0.001), had higher fasting glucose levels, insulin levels, and HOMA-IR levels (*P* < 0.001), and showed more severe lipid abnormalities (i.e., hyper-TC, hyper-TG, hyper-LDL, hyper-apoB, and hypo-apoA-I, *P* < 0.05). Furthermore, simple snorers had a higher prevalence of MetS (18.0% vs. 9.1%, respectively, *P* = 0.003), and among simple snorers, the percent of participants scored from 0 to 5 was 24.9%, 33.6%, 23.6%, 12.7%, 4.1%, and 1.2%, respectively; among controls, the percent was 31%, 40.6%, 19.3%, 6.4%, 2.7%, and 0, respectively. Simple snorers were more likely to be current smokers and alcohol drinkers (*P* < 0.001) (Table 1).

Compared to female simple snorers, males snored louder (higher VAS score, *P* < 0.001), were more obese (evidenced by higher BMI, NC, WC, HC, and WHR, *P* < 0.05), had higher systolic and diastolic blood pressure (*P* < 0.001), and had more severe dyslipidemia (i.e., higher TG, LDL, and apoB and lower HDL, apoA-I, and apoE, *P* < 0.05). In addition, males were more likely to be current smokers and alcohol drinkers than females (*P* < 0.001) (Table 2).

### 3.2. Prevalence of MetS

The prevalence rates of MetS and its components in our study are shown in Table 2. The prevalence rate of MetS was higher in simple snorers (18.0% vs. 9.1%, respectively, *P* = 0.003). Among its components, the prevalence rates of hypertension (29.3% vs. 18.7%, respectively, *P* = 0.004) and hyper-TG (25.9% vs. 12.8%, respectively, *P* < 0.001) were significantly higher in simple snorers than in nonsnorers.

Subgroup analysis showed that there were differences between simple snorers and the nonsnoring group in both male and female participants. Among male participants, simple snorers only had a higher prevalence rate of hypertension compared with nonsnorers (33.2% vs. 14.1%, *P* = 0.001). Among female participants, simple snorers had a higher prevalence rate of MetS (19.1% vs. 9.2%, *P* = 0.018) and higher prevalence rates of abdominal obesity (30.7% vs. 22.0%, *P* = 0.005) and hyper-TG (16.3% vs. 6.4%, *P* = 0.011) compared with nonsnorers (Table 3).

### 3.3. Association between Snoring and MetS

**Figure 2:**
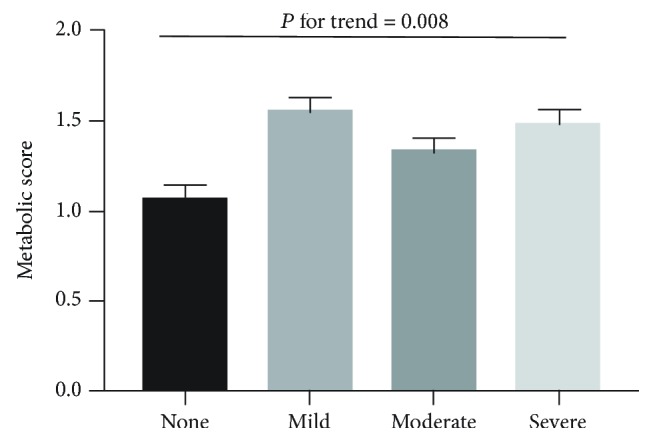
Adjusted mean metabolic scores across snoring severity. The data were adjusted for age, sex, smoking status, and alcohol consumption.

A significant linear trend was observed between snoring intensity and metabolic score (*P* for trend = 0.008) (Figure 2). Simple snoring was associated with MetS, even after adjusting for age, sex, smoking status, and alcohol consumption (OR = 2.328, 95% CI: 1.340–4.045). Gender stratification analysis showed that such association was only significant among female participants (OR = 2.382, 95% CI: 1.136–4.994) (Table 4).

Focusing on separate MetS components (Table 5), simple snoring was significantly associated with hypertension (OR = 1.730, 95% CI: 1.130–2.650) among all participants after adjusting for confounding factors, such as age, gender, smoking status, alcohol consumption, and other MetS components. However, the results of gender stratification analysis showed that this relationship was only obvious in men (OR = 3.493, 95% CI: 1.748–6.979).

Simple snoring was significantly associated with abdominal obesity (OR = 1.810, 95% CI: 1.063–3.083) after adjusting for confounding factors, and this relationship was only significant in women (OR = 2.306, 95% CI: 1.245–4.270). Simple snoring was also significantly associated with hyper-TG (OR = 1.814, 95% CI: 1.097–2.998), and gender stratification analysis showed that this relationship was only significant in women (OR = 2.803, 95% CI: 1.146–6.856) after multivariable adjustment.

The associations between simple snoring and hypo-HDL/hyperglycemia were not significant.

**Figure 3:**
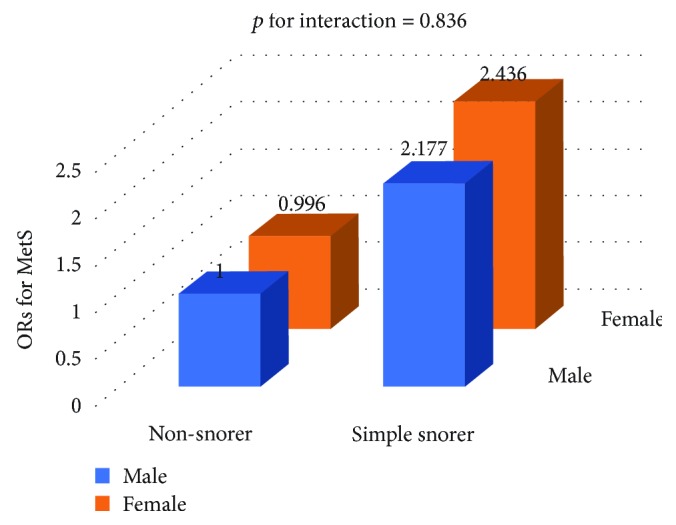
ORs for MetS according to gender and snoring status. ORs according to joint classification were adjusted for age, smoking status, and alcohol consumption.

No significant interaction between sex and snoring on MetS was observed through joint classification analysis (*P* for interaction = 0.836) (Figure 3).

## 4. Discussion

The present study indicated that simple snoring was associated with higher prevalence of MetS, and there was a positive linear trend for metabolic score across snoring severity after adjusting for multiple variables. Furthermore, we found that simple snoring was independently associated with MetS and that female snorers were more vulnerable to metabolic disorders. No interaction was observed between sex and simple snoring on MetS.

Previous studies have demonstrated a relationship between snoring and MetS, but did not exclude the impact of repeated apnea and hypoxia [10, 11, 20]. A similar relationship was still found after excluding OSA patients from the whole sample. Furthermore, some studies showed that associations between snoring and MetS/MetS components only existed among females [7, 13, 21–24]. In the present study, although men showed more typical symptoms, such as snoring, than women, the metabolic effects of snoring were even greater in women. Furthermore, simple snoring was significantly associated with MetS in female snorers but not in male snorers. After controlling for confounding factors, the association with abdominal obesity/hyper-TG was more obvious among women. However, no significant interaction was observed between sex and snoring in our study. The mechanism involved in this gender difference may be as follows. Firstly, approximately 7% of premenopausal women have polycystic ovary syndrome, show hyperandrogenism, and have increased vulnerability to sleep disorders and metabolic disorders [7, 21]. Secondly, the levels of sex hormone secretion are reduced in postmenopausal women, with the most pronounced changes seen in estrogen reduction. The hormone levels change from estrogen predominance to androgen predominance, followed by increased incidence of snoring and metabolic disorders. Thirdly, compared with men, women tend to accumulate less visceral fat, but there are fewer *α*-adrenergic receptors in visceral adipose tissue in men indicating higher rates of lipolysis [25]. Finally, there existed gene-by-sex interaction on hepatic steatosis and females could accumulate more hepatic TG than males genetically [26].

Obesity is considered a predisposing factor for severe snoring, whereas snoring may further promote the development of obesity [27]. Abdominal obesity is not only a component of MetS, but a promoting factor of other components of MetS, such as IR, resulting in more serious metabolic problems [28]. Thus, the relationships among snoring, obesity, and MetS are complex. Elmasry et al. [29] suggested that snoring and obesity lead to increased odds for diabetes, with obesity playing the leading role, whereas snoring further increases the odds for developing diabetes on the basis of obesity. Other studies [11, 29–31] have shown that snoring increases the odds for metabolic disturbances, but the effect of snoring may be significantly attenuated or even eliminated after adjusting for obesity-related indicators, such as BMI or WHR. Thus, obesity may play a leading role in the interaction between snoring and metabolic disorders by activating chronic inflammatory reactions, as well as adipokine disorders [11]. In this study, simple snorers were more obese, and simple snoring was significantly associated with abdominal obesity after adjustment, including for other MetS components.

The relationship between snoring and hypertension remains unclear. Lindberg et al. reported that snoring was an independent risk factor for hypertension in men younger than 50 years old, whereas snoring did not significantly affect hypertension in older adults [32]. Hu et al. reported that snorers had significantly higher systolic and diastolic blood pressure levels, and snoring was significantly associated with hypertension in women [23]. Bixler et al. reported that in both men and women, snoring was independently correlated with hypertension, which was more obvious in young and normal-weight participants [33]. Two related studies in the Korean population indicated that snoring significantly increased the prevalence of hypertension independent of obesity [20]. In contrast, Nieto et al. reported that AHI was significantly associated with hypertension, whereas the relationship between habitual snoring and hypertension was not obvious [34]. In the present study, simple snorers showed significantly higher prevalence rates of hypertension than nonsnorers, and simple snoring was an independent risk factor for hypertension, which was more obvious in men than in women. The main underlying mechanism may lie in estrogen, which could influence artery stiffness, inhibit vascular remodel, and modulate the renin-angiotensin aldosterone system [35].

Previous studies have also drawn inconsistent conclusions regarding the relationship between snoring and IR. Some researchers reported that snoring was associated with diabetes and insulin sensitivity [29, 36], whereas other studies showed that although snoring was more common in men, it significantly increased the odds for diabetes or impaired glucose tolerance only in women [13, 21, 24]. A recent meta-analysis concluded that there was a strong association between snoring and diabetes in women, but not in men [7]. The association between simple snoring and hyperglycemia was not significant in our study, but simple snorers showed higher fasting glucose and insulin levels as well as a tendency for higher IR than nonsnorers.

There have been few previous studies on the relationship between snoring and dyslipidemia. Shin et al. reported that as snoring frequency increased, TG levels increased and HDL levels decreased and that snoring was associated with hyper-TG and hypo-HDL, but the effect of snoring was significantly attenuated after adjusting for other confounding factors. Cho et al. reported that snorers had higher prevalence rates of hyper-TG and hypo-HDL, but snoring did not increase the odds for dyslipidemia after taking various confounding factors into consideration [20]. In our study, simple snorers had higher levels of TC, TG, LDL, and apoB, lower apoA-I levels, and higher prevalence of hyper-TG than nonsnorers. Furthermore, the components of dyslipidemia may differ between genders, e.g., males showed higher TG, LDL, and apoB and lower HDL, apoA-I, and apoE than females. However, after multivariable adjustment, simple snoring was significantly associated with increased odds for hyper-TG only among females.

The mechanisms underlying the relationship between simple snoring and MetS have not been clarified. Through direct mechanical injury to the endothelium and local initiation of proinflammatory response, snoring vibration transmission may accelerate the development of carotid atherosclerotic plaque and contribute to MetS [37–40]. The physiological disturbances caused by snoring increase the number of microarousals during sleep [22], which could disrupt the restorative value of sleep, increase the activity of the sympathetic nervous system, and have a harmful impact on the hypothalamic-pituitary-adrenal axis with consequent elevations in serum cortisol, ultimately contributing to metabolic dysfunction [13, 41–45].

To our knowledge, this is the first study to investigate the associations between MetS/MetS components and simple snoring excluding the impact of OSA. Our study showed that snoring without repeated apnea and hypoxia was significantly associated with increased odds for MetS, which requires more attention regarding hypertension among male snorers and abdominal obesity and hyper-TG among female snorers. Our study also had some limitations. First, although self-reported snoring and snoring reported by roommates were found to be reliable measures in epidemiological studies, as validated by all-night sleep recording [46, 47], these are subjective measures to evaluate snoring. Second, as the duration of snoring was not collected, its impact on MetS could not be assessed.

In conclusion, the present study investigated the relationships between simple snoring and MetS/MetS components and concluded that, after eliminating the effects of repeated apnea and hypoxia, simple snoring was still independently associated with increased odds for MetS, especially in women. Furthermore, it suggested that clinicians should implement interventions, such as suggesting low-fat diet, low-sugar diet, and increased exercise for simple snorers to prevent MetS.

## Figures and Tables

**Table 1:** Basic characteristics of nonsnorer participants and simple snorers.

	Nonsnorers (*n* = 187)	Simple snorers (*n* = 679)	*P* value
Age (years)	42.00 (33.00, 49.00)	36.00 (30.00, 46.00)	0.002
Male (%)	78 (41.7%)	428 (63.0%)	<0.001
BMI (kg/m^2^)	23.04 ± 3.11	24.15 ± 3.59	<0.001
NC (cm)	34.67 ± 3.29	36.80 ± 3.55	<0.001
WC (cm)	83.09 ± 10.09	86.99 ± 10.41	<0.001
HC (cm)	94.37 ± 7.07	97.10 ± 7.07	<0.001
WHR (cm)	0.88 ± 0.07	0.90 ± 0.07	0.011
SBP (mmHg)	119.19 ± 10.71	119.83 ± 14.39	0.573
DBP (mmHg)	76.55 ± 8.18	76.24 ± 9.72	0.687
FPG (mmol/L)	4.99 (4.62, 5.29)	5.05 (4.73, 5.37)	0.036
FINS (*μ*U/mL)	5.52 (4.08, 8.48)	7.85 (5.65, 11.30)	<0.001
HOMA-IR	1.20 (0.88, 1.90)	1.77 (1.23, 2.57)	<0.001
TC (mmol/L)	4.22 (3.49, 4.82)	4.37 (3.80, 4.96)	0.001
TG (mmol/L)	0.90 (0.63, 1.32)	1.18 (0.78, 1.72)	<0.001
HDL (mmol/L)	1.14 (0.96, 1.34)	1.09 (0.96, 1.29)	0.237
LDL (mmol/L)	2.39 (1.81, 2.93)	2.39 (1.81, 2.93)	<0.001
apoA-I (g/L)	1.14 (0.98, 1.29)	1.09 (0.96, 1.23)	0.023
apoB (g/L)	0.68 (0.57, 0.78)	0.76 (0.65, 0.88)	<0.001
apoE (mg/dL)	3.92 (3.24, 4.71)	3.90 (3.20, 4.79)	0.748
Lpa (mg/dL)	6.40 (3.75, 14.75)	8.10 (4.40, 16.80)	0.051
MetS, *n* (%)	17 (9.1)	122 (18)	0.003
Smokers, *n* (%)	15 (8.0)	149 (21.9)	<0.001
Alcohol drinkers, *n* (%)	16 (8.6)	198 (29.2)	<0.001

BMI: body mass index; NC: neck circumference; WC: waist circumference; HC: hip circumference; WHR: waist-to-hip ratio; SBP: systolic blood pressure; DBP: diastolic blood pressure; FBG: fasting blood glucose; FINS: fasting insulin; HOMA-IR: homeostasis model assessment of insulin resistance; TC: total cholesterol; TG: triglyceride; HDL: high-density lipoprotein cholesterol; LDL: low-density lipoprotein cholesterol; apoA-I: apolipoprotein A-I; apoB: apolipoprotein B; apoE: apolipoprotein E; Lpa: lipoprotein(a); MetS: metabolic syndrome.

**Table 2:** Basic characteristics of participants according to gender.

	Nonsnorers			Simple snorers		
	Male	Female	*P* value	Male	Female	*P* value
	(*n* = 78)	(*n* = 109)		(*n* = 428)	(*n* = 251)	
Age (years)	38.31 ± 12.64	42.16 ± 10.43	0.029	36.84 ± 11.42	40.60 ± 12.39	<0.001
BMI (kg/m^2^)	23.14 ± 3.08	22.96 ± 3.15	0.074	24.44 ± 3.22	23.66 ± 4.11	0.010
NC (cm)	37.14 ± 2.98	32.89 ± 2.18	<0.001	38.42 ± 2.73	34.04 ± 3.07	<0.001
WC (cm)	86.08 ± 11.37	80.95 ± 8.49	0.001	89.22 ± 9.27	83.18 ± 11.15	<0.001
HC (cm)	95.25 ± 7.25	93.74 ± 6.90	0.154	97.80 ± 6.68	95.89 ± 7.55	0.001
WHR (cm)	0.90 ± 0.08	0.86 ± 0.06	0.001	0.91 ± 0.06	0.87 ± 0.07	<0.001
SBP (mmHg)	118.15 ± 8.27	119.93 ± 12.14	0.237	121.78 ± 13.74	116.50 ± 14.89	<0.001
DBP (mmHg)	76.19 ± 8.23	76.81 ± 8.17	0.614	77.38 ± 9.57	74.28 ± 9.69	<0.001
FPG (mmol/L)	5.06 (4.55, 5.30)	4.97 (4.66, 5.26)	0.969	5.23 ± 0.88	5.12 ± 0.85	0.094
FINS (*μ*U/mL)	5.07 (3.42, 7.74)	5.87 (4.47, 8.59)	0.085	9.25 ± 5.75	10.28 ± 16.57	0.341
HOMA-IR	1.13 (0.76, 1.63)	1.29 (0.94, 2.03)	0.173	2.24 ± 1.89	2.43 ± 4.30	0.517
TC (mmol/L)	4.08 (3.39, 4.71)	4.25 (3.63, 4.85)	0.266	4.45 ± 0.84	4.40 ± 0.96	0.450
TG (mmol/L)	0.94 (0.67, 1.56)	0.87 (0.62, 1.19)	0.013	1.57 ± 1.26	1.23 ± 0.94	<0.001
HDL (mmol/L)	1.05 (0.88, 1.21)	1.21 (1.03, 1.41)	<0.001	1.08 ± 0.26	1.24 ± 0.28	<0.001
LDL (mmol/L)	2.41 (1.77, 2.99)	2.37 (1.90, 2.86)	0.934	2.79 ± 0.73	2.64 ± 0.83	0.016
apoA-I (g/L)	1.06 (0.92, 1.20)	1.22 (1.03, 1.34)	<0.001	1.07 ± 0.19	1.19 ± 0.23	<0.001
apoB (g/L)	0.68 (0.56, 0.78)	0.67 (0.58, 0.78)	0.833	0.78 ± 0.16	0.73 ± 0.18	<0.001
apoE (mg/dL)	3.62 (3.02, 4.25)	4.07 (3.45, 4.75)	0.160	4.04 ± 1.33	4.32 ± 1.40	0.013
Lpa (mg/dL)	6.10 (3.85, 11.55)	7.40 (3.70, 18.10)	0.051	13.76 ± 15.54	14.54 ± 18.06	0.578
MetS, *n* (%)	7 (9%)	10 (9.2%)	0.963	74 (17.3%)	48 (19.1%)	0.548
Smoking, *n* (%)	15 (19.2%)	0	<0.001	143 (33.4%)	6 (2.4%)	<0.001
Alcohol drinkers, *n* (%)	15 (19.2%)	1 (0.9%)	<0.001	177 (41.4%)	21 (8.4%)	<0.001

BMI: body mass index; NC: neck circumference; WC: waist circumference; HC: hip circumference; WHR: waist-to-hip ratio; SBP: systolic blood pressure; DBP: diastolic blood pressure; FBG: fasting blood glucose; FINS: fasting insulin; HOMA-IR: homeostasis model assessment of insulin resistance; TC: total cholesterol; TG: triglyceride; HDL: high-density lipoprotein cholesterol; LDL: low-density lipoprotein cholesterol; apoA-I: apolipoprotein A-I; apoB: apolipoprotein B; apoE: apolipoprotein E; Lpa: lipoprotein(a); MetS: metabolic syndrome.

**Table 3:** Prevalence of MetS in nonsnorers and simple snorers.

		Nonsnorers	Simple snorers	*P* value
Total	MetS	17 (9.1%)	122 (18.0%)	0.003
	Hypertension	35 (18.7%)	199 (29.3%)	0.004
	Abdominal obesity	23 (12.3%)	113 (16.6%)	0.148
	Hyper-TG	24 (12.8%)	176 (25.9%)	<0.001
	Hypo-HDL	101 (54.0%)	362 (53.3%)	0.866
	Hyperglycemia	21 (11.2%)	108 (15.9%)	0.112

Males	MetS	7 (9.0%)	74 (17.3%)	0.065
	Hypertension	11 (14.1%)	142 (33.2%)	0.001
	Abdominal obesity	5 (6.4%)	36 (8.4%)	0.551
	Hyper-TG	17 (21.8%)	135 (31.5%)	0.084
	Hypo-HDL	37 (47.4%)	207 (48.4%)	0.880
	Hyperglycemia	8 (10.3%)	72 (16.8%)	0.144

Females	MetS	10 (9.2%)	48 (19.1%)	0.018
	Hypertension	15 (19.0%)	57 (22.7%)	0.885
	Abdominal obesity	24 (22.0%)	77 (30.7%)	0.005
	Hyper-TG	7 (6.4%)	41 (16.3%)	0.011
	Hypo-HDL	64 (58.7%)	155 (61.8%)	0.587
	Hyperglycemia	13 (11.9%)	36 (14.3%)	0.539

MetS: metabolic syndrome; TG: triglyceride; HDL: high-density lipoprotein cholesterol.

**Table 4:** Odds ratios for MetS.

	Total		Males		Females	
	OR	95% CI	OR	95% CI	OR	95% CI
Unadjusted	2.190	(1.282–3.742)	2.120	(0.938–4.794)	2.341	(1.137–4.821)
Multivariable adjusted^∗^	2.328	(1.340–4.045)	1.615	(0.931–4.936)	2.382	(1.136–4.994)

^∗^Adjusted for age, sex, smoking status, and alcohol consumption.

**Table 5:** Odds ratios for MetS components.

		Total		Males		Females	
		OR	95% CI	OR	95% CI	OR	95% CI
Hypertension	Unadjusted	1.800	(1.203, 2.694)	3.024	(1.550, 5.902)	1.041	(0.606, 1.787)
	Multivariable adjusted^∗^	1.730	(1.130, 2.650)	3.493	(1.748, 6.979)	0.899	(0.501, 1.613)

Abdominal obesity	Unadjusted	1.424	(0.880, 2.303)	1.341	(0.509, 3.531)	2.237	(1.262, 3.965)
	Multivariable adjusted^∗^	1.810	(1.063, 3.083)	0.943	(0.335, 2.653)	2.306	(1.245, 4.270)

Hyper-TG	Unadjusted	2.376	(1.498, 3.771)	1.653	(0.930, 2.938)	2.845	(1.233, 6.562)
	Multivariable adjusted^∗^	1.814	(1.097, 2.998)	1.443	(0.774, 2.690)	2.803	(1.146, 6.856)

Hypo-HDL	Unadjusted	0.972	(0.703, 1.345)	1.038	(0.640, 1.683)	1.135	(0.718, 1.796)
	Multivariable adjusted^∗^	0.876	(0.615, 1.249)	0.808	(0.477, 1.368)	0.897	(0.551, 1.460)

Hyperglycemia	Unadjusted	1.495	(0.908, 2.461)	1.770	(0.816, 3.838)	1.236	(0.627, 2.437)
	Multivariable adjusted^∗^	1.217	(0.709, 2.092)	1.696	(0.736, 3.911)	0.899	(0.425, 1.899)

^∗^Adjusted for age, sex, smoking status, alcohol consumption, and other MetS components. TG: triglyceride; HDL: high-density lipoprotein cholesterol.

## Data Availability

The clinical data used to support the findings of this study are available from the corresponding authors upon request.
